# Focal boosted IMRT treatment of prostate cancer to 84 Gy in 28 fractions: preliminary clinical outcomes, toxicity, and dosimetry

**DOI:** 10.3389/fonc.2025.1577359

**Published:** 2025-05-20

**Authors:** Jacob Michael Hands, Michael J. Whalen, Shawn Haji-Momenian, Harold Frazier, Ramez Andrawis, Destie Provenzano, Julie E. Bauman, Fayez Estephan, Hamid Aghdam, Dongjun Chen, Sharad Goyal, Martin Ojong-Ntui, Yuan James Rao

**Affiliations:** ^1^ Radiation Oncology, George Washington (GW) University, Washington, DC, United States; ^2^ Urology, George Washington (GW) University, Washington, DC, United States; ^3^ Radiology, George Washington (GW) University, Washington, DC, United States; ^4^ Medical Oncology, George Washington (GW) University, Washington, DC, United States

**Keywords:** radiation oncologist, IMRT, SBRT, radiotoxicity, dosimetry

## Abstract

**Introduction:**

The FLAME trial reported that focal boosting of prostate tumors up to 95 Gy in 35 fractions improves biochemical control (disease-free survival). However, this treatment (regimen) is not commonly used in the United States. We investigated a focally boosted treatment of 84 Gy in 28 fractions (EQD2–108 Gy, BED 252 Gy).

**Methods:**

We retrospectively evaluated men with unfavorable intermediate-risk (uIR) and high-risk (HR) prostate cancer treated with focal boost intensity-modulated radiotherapy (IMRT) between 2019 and 2022. The dose levels were 84 Gy to the gross tumor volume (GTV), 70 Gy to the prostate and proximal seminal vesicles, and an optional 50.4 Gy to elective pelvic lymph nodes (all 28 fractions). The treatment planning goal was to cover 95% of the GTV at 84 Gy, and also meet the target and normal tissue dosimetry criteria of the hypofractionated treatment arm of NRG-GU005. Volume-modulated arc therapy was used for treatment delivery. Androgen deprivation therapy (ADT) was given at the discretion of the treating physician.

**Results:**

In total, 20 men were included in the analysis, 2 (10%) with uIR and 18 (90%) with HR. Six (30%) tumors were GG2, three (15%) GG3, seven (30%) GG4, and four (20%) GG5. There were 13 (65%) stage cT1, 4 (20%) cT2, and 3 (15%) cT3 tumors. One (5%) patient received short-term ADT, 18 (95%) long-term ADT, and 1 (5%) refused ADT. Moreover, 18 (90%) men received elective pelvic nodal radiation. The mean baseline Prostate specific antigen (PSA) was 25.1 ng/mL (range 4.2–73.4). The median baseline International Prostate Symptom Score (IPSS) was 11.1 (IQR 4.5–12). Four patients had severe baseline urinary symptoms (IPSS ≥20). The mean baseline prostate volume was 57.4 cc (range 26.8–198.3). The mean volume of the 84 Gy boost target was 7.1 cc (range 2.3–15.0) and the mean proportion of the prostate boosted was 14.8% (range 2%–47%). Patients met all per-protocol normal tissue criteria of NRG-GU005, except for bladder D0.03cc, with a reported mean of 79.2 (≤73.5 Gy). At a median follow-up of 42 months (range 18–63), no patients had developed recurrence, metastasis, or death from prostate cancer. One patient died at 18 months from unrelated metastatic colorectal cancer. Acute grade 1–2 genitourinary (GU) toxicity occurred in 13 (65%) patients, and acute grade 1–2 gastrointestinal (GI) toxicity occurred in 4 (20%) patients. No patients developed grade 3+ acute or late GU or GI toxicity.

**Conclusion:**

A novel 28-fraction focal boosted IMRT treatment is feasible and has an acceptable preliminary toxicity profile. Oncologic results are promising but require longer follow up and prospective study.

## Introduction

External beam radiotherapy (EBRT) remains a common treatment for intermediate and high-risk prostate cancer. However, local disease recurrence within the prostate remains an important cause of treatment failure, especially in unfavorable intermediate (uIR) and high-risk (HR) patients (1–4). High-dose radiotherapy techniques such as whole-gland dose escalation using EBRT or brachytherapy may improve biochemical progression-free survival (bPFS), though some studies have suggested increased toxicity (5–20) Novel EBRT methods of focal dose escalation of radiotherapy might result in iso-toxic treatments while improving biochemical disease control (21, 22).

At present, the American Society for Radiation Oncology (ASTRO) guidelines recommend dose-escalated intensity-modulated radiotherapy (IMRT) regimens or additional brachytherapy boost for the treatment of intermediate and high-risk prostate cancer (23–25). Specifically, with respect to dose, most radiation oncologists would consider a dose of approximately 78–80 Gy or its biological equivalent to be standard of care (26). The potential toxicity of higher dose-escalation (beyond 80 Gy) has been explored, though the preponderance of authors have documented the safety of regimens greater than 80 Gy with several prior authors noting the safety of “ultrahigh” dose-escalation therapies up to 86 Gy. Rosenbrook et al. (25) reported an excellent toxicity profile yielding no Grade 3 or greater adverse events using an 84 Gy dose-escalated therapy delivered via volume-modulated arc therapy (VMAT) (14, 15, 27). Similarly, trials assessing moderate hypofractionation (typically >2.5 Gy/fraction) have found that moderate and even ultra-hypofractionation (typically greater than 5 Gy/fx) have not resulted in any Grade 4 toxicity, though there may exist differences in rates of acute and late genitourinary (GU) and gastrointestinal (GI) toxicities (12, 13). Additionally, GETUG-AFU 18, a randomized trial on 80 vs. 70 Gy for high-risk prostate cancer, recently reported a 10-year PFS of 83.6% vs. 72.2%, respectively (P = .0005). The 10-year rate of cancer-specific survival was 95.6% with 80 Gy and 90.0% with 70 Gy (P = .0090). Overall survival (OS) was also improved, with a hazard ratio of 0.61 (28). This study was the first randomized trial demonstrating improved OS with dose escalation in prostate cancer and likely will be a landmark establishing 80 Gy or higher as the target dose equivalent in prostate cancer radiotherapy. The hypo-FLAME trial (2024) tested an ultra-hypo fractionated stereotactic body radiation therapy (SBRT) regimen of 35 Gy in five sessions with an integrated boost up to 50 Gy over 25 days in men with intermediate and high prostate cancer risk over 5 years and documented a 93% biochemical disease-free survival (bDFS) and GU and GI toxicity risk of 12% and 4%, respectively (29). In a 2023 review of 35 trials and 34 planning studies, focal boosted therapy was generally associated with no significant difference in Common Terminology Criteria for Adverse Events (CTCAE)-defined cumulative toxicities, with acute GU (32.8%) late GU (19.3%), acute GI (14.4%), and late GI (10.5%) ≥ Grade 2 toxicities comprising the majority of adverse events, with a correlation identified between lower-risk patients and a lower proportion of toxicity (30).

In this context, it is desirable to accomplish dose escalation while not increasing the overall toxicity of treatment. One way in which this might be done is with ‘focal boosting.’ The Focal Lesion Ablative Microboost in Prostate Cancer (FLAME) trial demonstrated that 77 Gy (35 fractions) to the whole gland with a focal boost up to 95 Gy, delivered to the macroscopic tumor volume, resulted in improved bDFS as compared with a non-boosted IMRT therapy (30, 31). While the FLAME trial demonstrated compelling evidence of improved clinical outcomes using a focal boost of up to 95 Gy in 35 fractions, this treatment regimen is not commonly used in clinical practice in the United States, due to a preference for 40–44 fractions for conventional fractionation, or 20–28 fractions for moderate hypofractionation (32). In the present study, we investigated the safety and preliminary clinical outcomes of a focally boosted IMRT treatment of 84 Gy given in 28 fractions to the whole macroscopic tumor volume using VMAT.

## Methods

We retrospectively evaluated the medical records of men with uIR or HR prostate cancer who received focal boosted IMRT treatment between 2019 and 2022 (IRB# NCR191470) at George Washington University Medical Center. We included patients with T1c through to T3a disease. Patients with seminal vesicle invasion were not included in this study. All men had multiparametric MRI (mpMRI)-visible prostate cancer and desired hypofractionated radiation in 28 fractions. Androgen deprivation therapy (ADT) was given at the discretion of the treating physician and was segmented by short-term (4–6 months) and long-term ADT (18–36 months). All patients received fiducial markers. A hydrogel spacer was placed for all patients except for those with extra-prostatic extension. Gleason group, International Prostate Symptom Score (IPSS), PSA, and other clinical variables were recorded. There was no specific limitation on the size or number of intraprostatic targets for eligibility for this regimen. However, an empirical limit of less than 50% of the total prostate volume was imposed in order to limit toxicity.

For radiation treatment planning, the dose levels were 84 Gy to the gross tumor volume (GTV) as defined on mpMRI (T2W and ADC) with no added margin, 70 Gy to the prostate and proximal seminal vesicles, and an optional 50.4 Gy to elective pelvic lymph nodes (all in 28 fractions). We verified that each GTV corresponded to a location of positive biopsy from the pathology report. All positive lesions were included in the GTV regardless of Gleason grade. In patients with extra-prostatic extension (EPE), the GTV boost volume was allowed to extend minimally outside the prostate, corresponding to the region of EPE, but the GTV was confined within the prostate for patients without EPE. In patients with EPE, all of the tumor outside of the prostate was covered in the GTV boost volume. There was no pre-specified distance or volume of EPE that was considered ineligible for treatment. However, patients with seminal vesicle involvement were not treated with this method. With an alpha/beta ratio of 1.5 for prostate cancer, the biological equivalent doses (BED1.5) were 252 Gy and 187 Gy for the GTV and prostate, respectively.

The treatment planning goal was to cover 95% of the GTV at 84 Gy, and also meet the target and normal tissue dosimetry criteria of the hypofractionated treatment arm of NRG-GU005 (**Table 1**) (29, 33). These criteria were selected because NRG-GU005 was the largest and most recent multi-institutional randomized trial with a moderately hypofractionated treatment arm in 28 fractions in the United States. Treatment plans were generated on Raystation software using a VMAT method. Treatments generally used two coplanar arcs. The linear accelerator used was a Varian TrueBeam with micro-multileaf collimators. Patients were treated with image-guided radiation therapy (IGRT) daily, with matching by the fiducial markers.

**Table 1:** Dose constraints for the hypofractionated IMRT arm of NRG GU-005.

Name of structure	Dosimetric parameter	Constraint
Rectum	D15%	≤70 Gy
	D25%	≤65 Gy
	D30%	≤50 Gy
	D50%	≤38 Gy
Bladder	D0.03cc	≤73.5 Gy
	D30%	≤50 Gy
	D50%	≤38 Gy
	D90%	≤15 Gy
Bowel	D0.03	≤45 Gy

All GU and GI toxicities were recorded and assessed according to the CTCAE v5 with Grade 2 GU being implied by moderate disturbances pertaining to dysuria, frequency, urgency, incontinence, obstruction, and retention, and Grade 2 GI being implied by hemorrhage, ulceration, obstruction, stenosis, diarrhea, nausea or vomiting, and bloating. AE data were collected in an institutional prospective registry. Patients received routine follow-up with PSA testing every 3–6 months after treatment. Acute toxicity was reported if occurred during treatment up to 3 months. Late toxicity was defined as 3 months post-treatment to the last date of follow-up. Oncologic outcomes reported included bPFS and OS.

Welch’s t-test was performed to identify the likelihood of any variable being associated with an adverse event rate during treatment. Statistics were calculated using STATA v18 (College, TX).

## Results

In total, 20 men were included in the study, with 2 (10%) of them uIR and 18 (90%) HR. Furthermore, 65% (13/20) of patients were Black or African American, 30% (6/20) White, and 5% (1/20) Hispanic. With respect to the Gleason grade groups, six (30%) tumors were GG2, three (15%) were GG3, seven (35%) were GG4, and four (20%) were GG5. There were 13 (65%) stage cT1, 4 (20%) cT2, and 3 (15%) cT3a tumors. One (5%) patient received short-term ADT, 18 (95%) received long-term ADT, and 1 (5%) refused ADT. Additional demographic, tumor, and treatment variables are provided in **Table 2**.

**Table 2:** Demographics.

N	20
Age	71.750 (6.365)
Race	
AA	13 (65.0%)
H	1 (5.0%)
W	6 (30.0%)
PSA	25.112 (18.110)
Baseline IPSS	11.050 (9.902)
T stage	
T1c	13 (65.0%)
T2a	1 (5.0%)
T2b	2 (10.0%)
T2c	1 (5.0%)
T3a	3 (15.0%)
Gleason score	
7	9 (45.0%)
8	7 (35.0%)
9	4 (20.0%)
Nodes treated	
No	2 (10.0%)
Yes	18 (90.0%)
Risk group	
HR	18 (95.0%)
LR	2 (10.0%)
Prostate	
MRI visible lesions	
1	10 (50.0%)
2	6 (30.0%)
3	3 (15.0%)
4	1 (5.0%)
Location	
PZ	14 (70.0%)
PZ TZ	3 (15.0%)
TZ	2 (10.0%)
TZ, CZ	1 (5.0%)

The mean baseline PSA was 25.1 (range 4.2–73.4). The median baseline IPSS score was 11.1 (IQR 4.5–12); four patients had severe baseline urinary symptoms (IPSS ≥20). The mean baseline prostate volume was 57.4 cc (range 26.8–198.3). The mean volume of the 84 Gy boost target was 7.1 cc (range 2.3–15.0) and the mean proportion of the prostate boosted was 14.8% (range 2%–47%). There were 10 (50%) men with one boost target, 6 (30%) with two, 3 (15%) with three, and 1 (5%) had four boost targets. Targets were located in peripheral zone (85%), transition zone (30%), and central zone (5%). Furthermore, 18 patients received elective nodal irradiation. Patients met all rectum and bladder per-protocol normal tissue criteria of NRG-GU005, except for bladder D0.03cc. The metric of D0.03cc ≤73.5 Gy was exceeded by all 20 patients (**Table 3**).

**Table 3:** Dosimetric data.

Variable	Mean	Median	SD	IQR	Minimum	Maximum
Rectum D50	3492.1	3466.5	349.7482	357.5	2720	4330
Rectum D30	4223.45	4215	440.9477	597.5	3366	5156
Rectum D2cc	6607.75	6953.5	765.4951	1180.5	4992	7494
Rectum D25	4469.4	4417	450.5244	682.5	3566	5284
Rectum D15	5118.6	5090	528.0013	705	4096	6156
Rectum D003cc	7857.3	7755	515.0429	963.5	6939	8710
Ptv_8400 D99	8239.45	8237	46.23222	35.5	8120	8374
Ptv_8400 D95	8402.45	8400	10.72368	0	8400	8448
Ptv_8400 D50	8779.65	8780.5	65.83494	95	8622	8912
Ptv_7000 D99	6626.15	6656	299.7449	227.5	5703	7122
Ptv_7000 D95	6994.5	7026.5	173.3173	139.5	6608	7291
Ptv_7000 D50	7486.6	7462	88.15859	112.5	7346	7682
Penilebulb D50	1027.85	728	866.1502	593	329	3935
Bladder D90	1966	1968.5	812.6828	381	187	4339
Bladder D50	3770.15	3778.5	981.3821	527	1024	5747
Bladder D30	4989.45	4982.5	761.6909	374.5	2721	6666
Bladder D2cc	7359.26	7445	376.1031	368.8	6120	7909
Bladder D15	5782.9	5599.5	829.0195	836	3788	7253
Bladder D003cc	7922.5	7802.5	388.0945	500	7487	8937

Regarding bowel dose, GU005 specified bowel D0.03cc ≤ 45 Gy, with < 50 Gy as an acceptable variation. However, elective nodal radiotherapy was not allowed in NRG-GU005 so this constraint is likely too stringent for patients receiving elective nodal radiation to a dose of 50.4 Gy. Among the 18 patients who received elective nodal radiotherapy in this cohort, the mean D0.03cc bowel dose (contoured as a “bowel bag”) was 50.3 Gy (range 39.1–55.6). In our institution, we have typically constrained the D0.03cc bowel dose to less than 55 Gy with this treatment regimen, and this metric was met in 17/18 (94%) patients who received elective nodal radiation. In the POP-RT trial of elective nodal radiation to 50 Gy, the bowel constraint was V55 Gy <14 cc, and this was met in all patients in this cohort (34).

**Figure 1:**
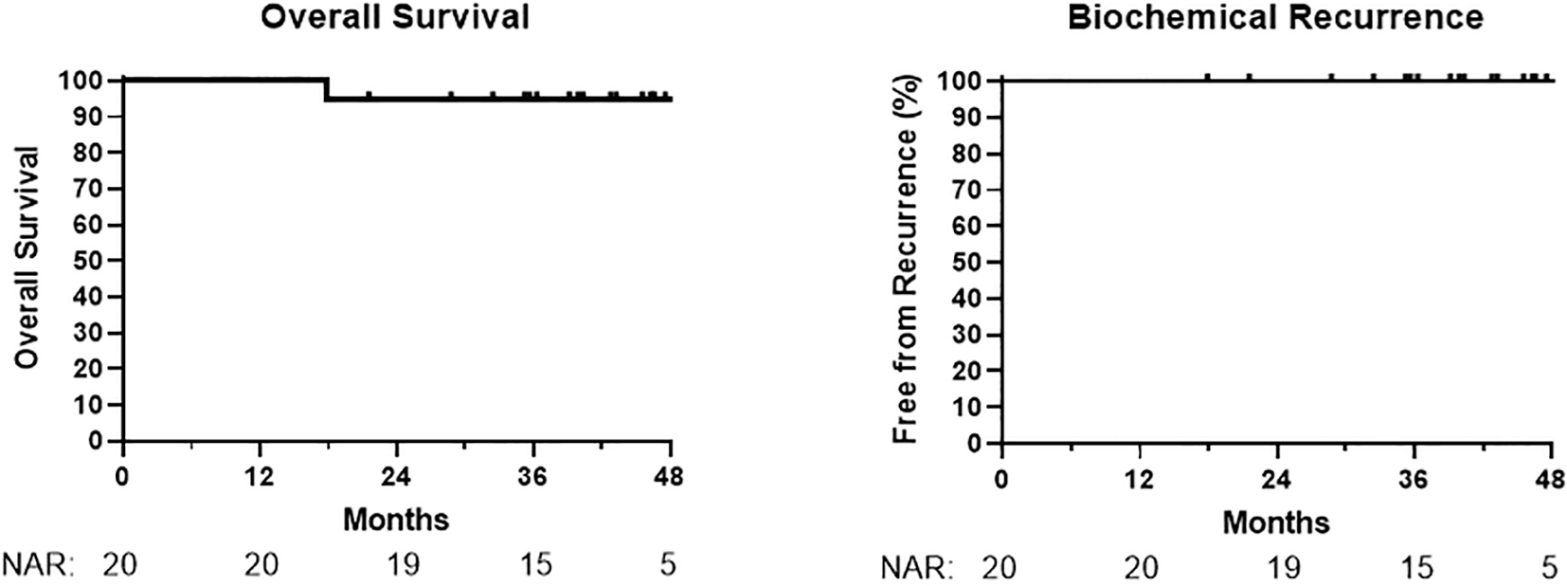
Overall survival and biochemical recurrence.

At a median follow-up time of 42 months (range 18–63), no patients had developed biochemical recurrence, metastasis, or death from prostate cancer (**Figure 1**). One patient died at 18 months from metastatic colorectal cancer, unrelated to prostate cancer treatment. A patient who refused ADT had a PSA increase of 2.2 ng/mL at 14 months (from 1.5 ng/mL to 3.7 ng/mL); PSA values decreased subsequently in this patient without additional treatment.

Acute grade 1–2 GU toxicity occurred in 13 (65%) patients, and acute grade 1–2 GI toxicity occurred in 4 (20%) patients. No patients developed grade 3+ acute or late GU or GI toxicity. Only one late toxicity event was reported and it was associated with late grade 1 nocturia. Acute toxicity events included one case each (5%) of grade 1 dysuria, diarrhea, photodermatitis, and fatigue, one case of (5%) grade 2 photodermatitis, and two cases of (10%) grade 2 dysuria. Both patients with grade 2 dysuria required a temporary Foley catheter for obstruction during RT, and both had IPSS >20 at baseline.

PTV8400D50 and the percentage of prostate boosted were independently associated with an increased likelihood of any grade adverse event (p= 0.01 and p= <0.001, respectively, **Supplementary Tables S2** and **S3**). No other baseline variables or treatment parameters were associated with the likelihood of an adverse event, including prostate volume and baseline IPSS.

## Discussion

In our study, we established the preliminary feasibility of a focal boosted IMRT regimen in 28 fractions that treats the MRI-defined prostate lesion to 84 Gy, the entire prostate to 70 Gy, and the elective pelvic lymph nodes to 50.4 Gy. This is an interesting result because a 28-fraction regimen is familiar to radiation oncologists practicing in the United States, and was also the standard of care treatment arm of NRG-GU005. In this retrospective analysis, we report the dosimetric feasibility and tolerability of this regimen, which modifies the standard 70 Gy in a 28-fraction IMRT regimen to include a focal boost and optional lymph node coverage. Additionally, early biochemical outcomes are promising at a median follow-up of 42 months, although long-term follow-up is needed.

Dosimetrically, we established the feasibility of a three-tiered integrated boost treatment design of 50.4 Gy to the elective pelvic lymph nodes, 70 Gy to the prostate, and 84 Gy to the focal boost lesions. The BED of 252 used in this regimen is slightly lower than that of FLAME but is greater than the previously identified necessary BED of 200 Gy for high-dose curative intent (28). We were able to meet all the dosimetric criteria of NRG-GU005 for all patients, except for bladder D0.03cc ≤73.5 Gy, which was exceeded by all 20 patients. However, this did not appear to be associated with excess GU toxicity. Indeed, we observed no Grade 3 or greater toxicity. Neither prostate volume nor IPSS demonstrated significant associations with adverse event rate, while the percentage of the prostate boosted was significantly associated with toxicity. This suggests that focal boosting may be an appropriate treatment regardless of prostate size, yet the proportion of the prostate treated with the focal boost may be considered in patient selection and warrants further investigation to optimize this volume threshold.

Oncologic outcomes in our cohort are promising at this time, as there have been no biochemical recurrence events. In the FLAME study, the focal boost arm had no recurrence events for approximately 2 years and our cohort appears to be matching this. However, nearly all patients in our cohort are receiving long-term ADT. Approximately twice the median follow-up to >5 years is desirable for future reporting.

The limitations of this study included the absence of prospective design, lack of contemporaneous comparator, small sample size, single institution, and a relatively modest follow-up time in addition to the single center and the retrospective design. This patient cohort had a large proportion of African American men (65%), which may limit generalizability to other populations, however, it is reassuring that focal boost appears to be safe and efficacious in this patient population.

Additionally, reporting of late toxicity may have been limited due to the retrospective nature of the study. Prospective studies with a larger sample size and longer follow-up are warranted. Furthermore, the implications of focal boost for all mpMRI-visible carcinoma vs. targeting regions of interest (ROIs) with clinically significant prostate cancer (i.e., GG2 or higher) remains to be explored. This aim is especially important given the demonstrable correlation between PTV8400D50 and the percentage of boost and the incidence of toxicity in our cohort. Overall, we propose that this 28-fraction regimen warrants prospective investigation as a shorter and more familiar alternative for U.S. practitioners compared to that presented in FLAME.

## Conclusion

This retrospective study reported safety and preliminary outcomes associated with an 84 Gy focal boost IMRT regimen in 28 fractions. This regimen was feasible and associated with an acceptable safety profile. No patients developed recurrence after a median follow-up of 42 months, although a longer follow-up is needed.

## Data Availability

The raw data supporting the conclusions of this article will be made available by the authors, without undue reservation.

## Supplementary material

Supplementary Table 1 Patient level data of patients in this study.

Supplementary Table 2 Linear regression of dosimetric variables against toxicity.

Supplementary Table 3 Additional tests of statistical correlation between dosimetric and clinical variables and toxicity. PTV_8400D50, any GU or GI event, two-sample t test with unequal variances.
