# Supplementary material for: Focal boosted IMRT treatment of prostate cancer to 84 Gy in 28 fractions: preliminary clinical outcomes, toxicity, and dosimetry
Source: Front Oncol. 2025 May 20;15:1577359. doi: 10.3389/fonc.2025.1577359 (PMC12129977; doi:10.3389/fonc.2025.1577359)

Supplemental Tables

Table S1 – Patient Level Data of Patients in this Study

| Age | Race | TStage | Hormone Therapy Agent | Hormone Therapy Duration | RiskGroup |
| --- | --- | --- | --- | --- | --- |
| 63 | aa | T1c | leuprolide | long term | HR |
| 75 | w | T1c | relugolix | long term | HR |
| 83 | aa | T3a | relugolix | long term | HR |
| 63 | aa | T3a | leuprolide | long term | HR |
| 76 | w | T1c | relugolix | long term | HR |
| 67 | aa | T2c | leuprolide | long term | HR |
| 70 | w | T1c | Declined ADT | Declined ADT | uIR |
| 68 | aa | T1c | leuprolide | long term | HR |
| 75 | h | T2b | leuprolide | long term | HR |
| 67 | aa | T1c | leuprolide | long term | uIR |
| 70 | aa | T2b | leuprolide | long term | HR |
| 69 | aa | T1c | leuprolide | long term | HR |
| 78 | w | T1c | relugolix | long term | HR |
| 79 | aa | T2a | leuprolide | long term | HR |
| 74 | w | T1c | relugolix | short term | HR |
| 69 | aa | T1c | leuprolide | long term | HR |
| 84 | w | T1c | relugolix | long term | HR |
| 70 | aa | T3a | leuprolide | long term | HR |
| 74 | aa | T1c | relugolix | long term | HR |
| 61 | aa | T1c | leuprolide | long term | HR |

Table S2: Linear regression of dosimetric variables against toxicity

|  |  | | |
| --- | --- | --- | --- |
|  | No AEs | Grade 1 or 2 | p-value |
| N | 6 (30.0%) | 14 (70.0%) |  |
| PTV_8400 - D95% | 8,400.167 (0.408) | 8,403.429 (12.829) | 0.548 |
| PTV_8400 - D99% | 8,226.500 (21.473) | 8,245.000 (53.254) | 0.427 |
| PTV_8400 - D50% | 8,727.167 (64.011) | 8,802.143 (54.246) | 0.015* |
| PTV_7000 - D95% | 6,948.000 (235.209) | 7,014.429 (145.599) | 0.447 |
| PTV_7000 - D99% | 6,600.167 (262.177) | 6,637.286 (323.160) | 0.807 |
| PTV_7000 - D50% | 7,458.833 (116.090) | 7,498.500 (75.281) | 0.371 |
| Rectum - D0.03 cc | 7,881.500 (576.493) | 7,846.929 (509.402) | 0.895 |
| Rectum - D2 cc | 6,639.917 (839.360) | 6,593.964 (764.711) | 0.906 |
| Rectum - D15% | 4,800.167 (393.280) | 5,255.071 (530.198) | 0.076 |
| Rectum - D25% | 4,295.500 (441.135) | 4,543.929 (449.295) | 0.270 |
| Rectum - D30% | 4,095.833 (451.190) | 4,278.143 (441.745) | 0.412 |
| Rectum - D50% | 3,409.167 (391.637) | 3,527.643 (339.490) | 0.502 |
| Bladder - D0.03 cc | 7,878.500 (260.963) | 7,941.357 (438.934) | 0.750 |
| Bladder - D2cc | 7,462.100 (188.454) | 7,315.186 (431.401) | 0.438 |
| Bladder - D15% | 5,578.667 (1,113.752) | 5,870.429 (707.023) | 0.486 |
| Bladder - D30% | 4,934.500 (1,265.430) | 5,013.000 (479.651) | 0.839 |
| Bladder - D50% | 3,830.333 (1,545.303) | 3,744.357 (697.702) | 0.863 |
| Bladder - D90% | 1,823.833 (807.208) | 2,026.929 (837.462) | 0.622 |
| Penile Bulb D50% | 967.833 (686.312) | 1,053.571 (955.475) | 0.846 |

Table S3 – **Additional tests of statistical correlation between dosimetric and clinical variables with toxicity.**

PTV_8400D50, Any GU or GI Event, Two-sample t test with unequal variances

| Group | Observations | Mean | Std Error | Std Dev | [95% CI] |
| --- | --- | --- | --- | --- | --- |
| No AEs | 6 | 8727.167 | 26.13225 | 64.01068 | 8659.992 – 8794.342 |
| Grade 1 or 2 | 14 | 8802.143 | 14.49777 | 54.24568 | 8770.822 – 8833.463 |
| t= -2.508 | Pr(T<t) = **0.0161** |  |  |  |  |

Percent of Boost, Any GU or GI Event, Two-sample t test with unequal variances

| Group | Observations | Mean | Std Error | Std Dev | [95% CI] |
| --- | --- | --- | --- | --- | --- |
| No AEs | 6 | 0.067 | .0128 | .031 | .0337026 .0996308 |
| Grade 1 or 2 | 14 | .1821429 | .0265441 | .0993191 | .1247977 .239488 |
| t = -3.9172 | Pr(T<t) <**0.001** |  |  |  |  |

IPSS, Any GU or GI Event, Two-sample t test with unequal variances

| Group | Observations | Mean | | Std Error | Std Dev | [95% CI] |
| --- | --- | --- | --- | --- | --- | --- |
| No AEs | 6 | 8.166667 | | 3.5449 | 3.545 | 4.44647 11.88686 |
| Grade 1 or 2 | 14 | 12.28571 | | 3.08208 | 11.53209 | 5.627286 18.94414 |
| t = -1.2097 | **Pr(T < t) = 0.1209** | |  |  |  |  |

Volume prostate, Any GU or GI Event, Two-sample t test with unequal variances

| Group | Observations | Mean | Std Error | Std Dev | [95% CI] |
| --- | --- | --- | --- | --- | --- |
| No AEs | 6 | 61.03 | 9.954 | 24.38296 | 35.44165 86.61835 |
| Grade 1 or 2 | 14 | 55.85143 | 11.43162 | 42.77322 | 31.15491 80.54795 |
| t = 0.3416 | **Pr(T < t) =0.6318** |  |  |  |  |

Supplement Figure S1


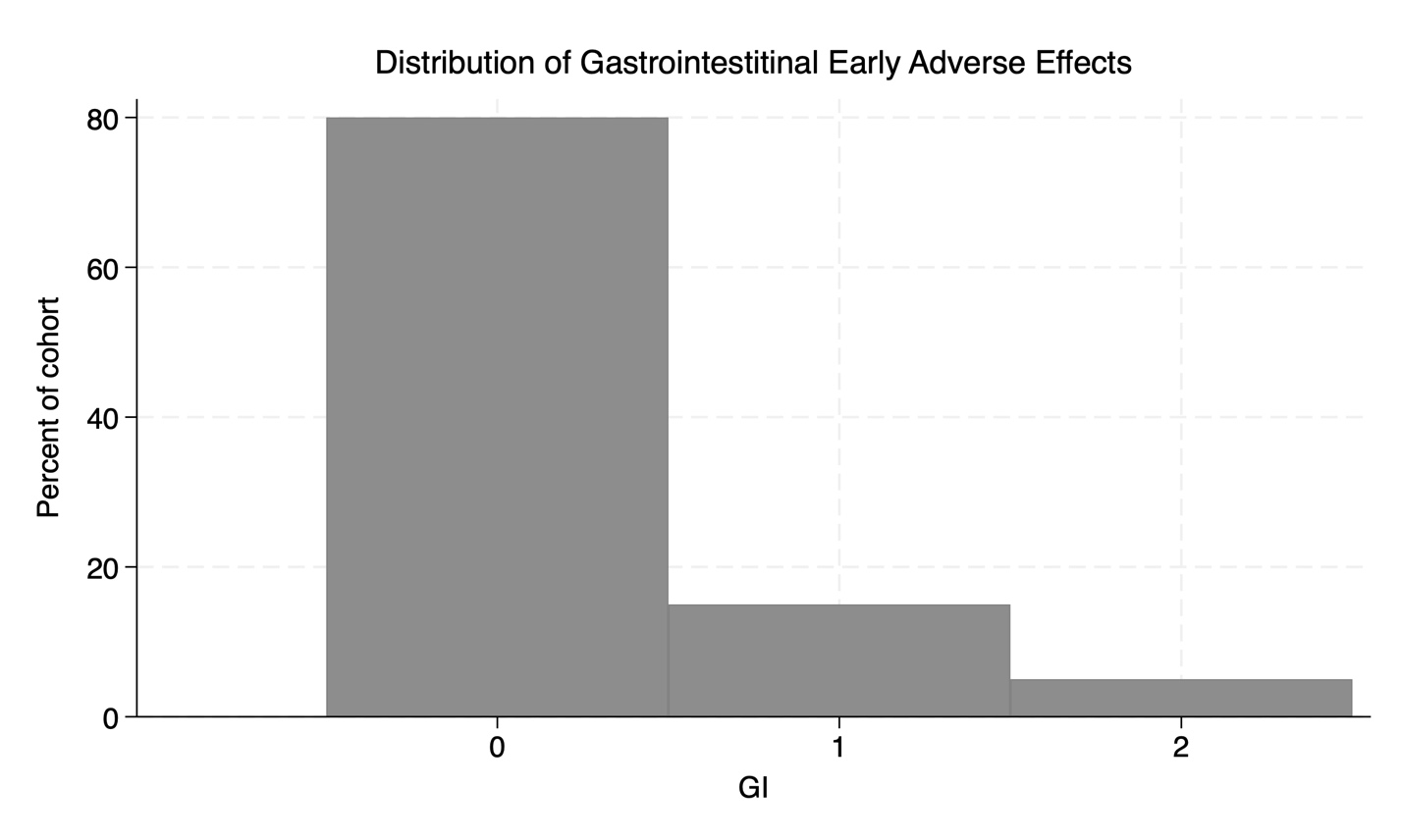


Supplement Figure S2


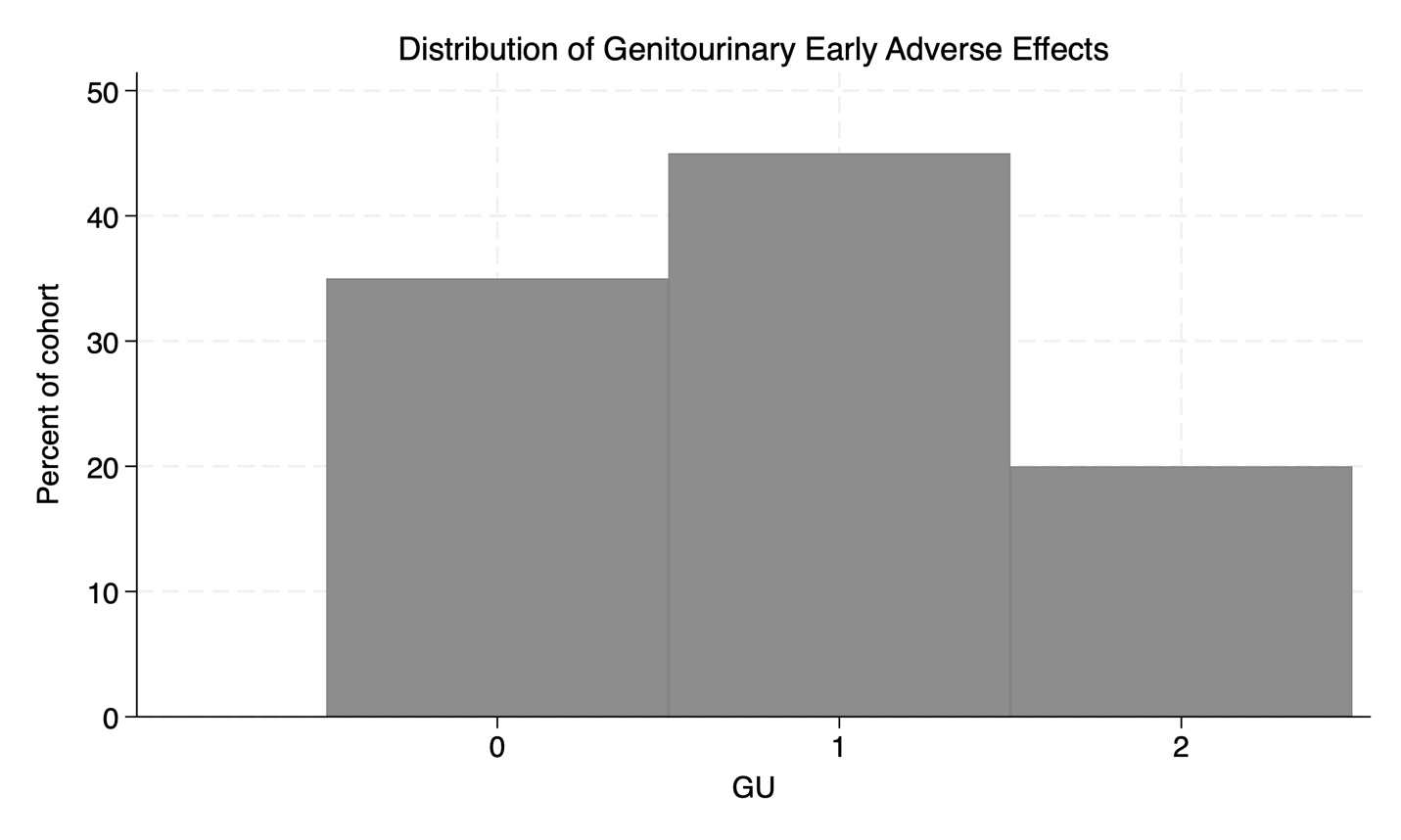


Supplement Figure S3


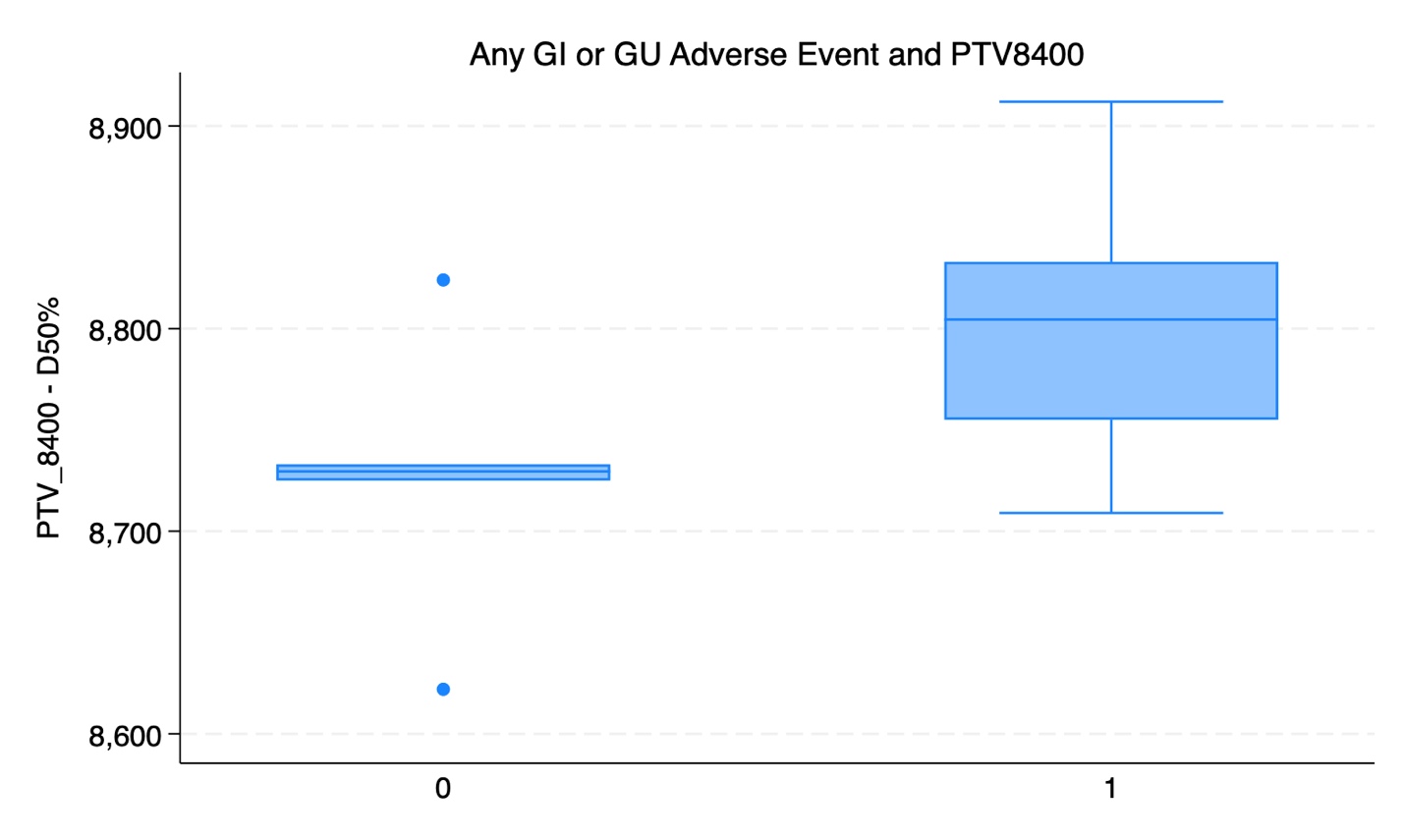

Supplement: Supplementary Table 1 — Patient level data of patients in this study. [file DataSheet1.docx]
